# En Bloc Spondylectomy for T11 Metastasis of Nasal Adenocarcinoma: A Case Report

**DOI:** 10.7759/cureus.101215

**Published:** 2026-01-10

**Authors:** Ajay Krishnan, Mikeson Panthackel, Shivanand C Mayi, Ravi Ranjan Rai, Mirant B Dave, Arjit Vashishtha, Mahesh Sagar, Yogenkumar Adodariya, Saurabh S Kulkarni, Amritesh Singh, Preety Krishnan, Bharat R Dave

**Affiliations:** 1 Spine Surgery, Bhavnagar Institute of Medical Sciences (BIMS), Bhavnagar, IND; 2 Spine Surgery, Stavya Spine Hospital and Research Institute, Ahmedabad, IND; 3 Orthopedics, Mahatma Gandhi Medical College and Research Institute, Aurangabad, IND; 4 Orthopedics, Geetanjali Medical College and Hospital, Udaipur, IND; 5 Orthopedics, University College of Medical Sciences and Guru Teg Bahadur Hospital, Delhi, IND; 6 Radiology, Stavya Spine Hospital and Research Institute, Ahmedabad, IND

**Keywords:** en bloc resection, spine metastasis, total spondylectomy, tumour, wbb classification

## Abstract

En bloc spondylectomy resection (EBR) has emerged as a potential option for achieving tumor-free margins and enhancing long-term outcomes in select cases of solitary spinal metastases, though the role of this treatment in metastatic spine disease remains debated. We present the case of a 49-year-old female with a prior history of adenoid cystic carcinoma of the nose who developed back pain two years later. Positron emission tomography-computed tomography (PET-CT) revealed a solitary T11 lesion, and a CT-guided biopsy confirmed metastasis. After meticulous surgical planning guided by the Weinstein-Boriani-Biagini (WBB) classification, an EBR of T11 was performed using a single posterior approach involving an ultrasonic bone scalpel, three-dimensional CT navigation, and intraoperative neuro-monitoring, followed by posterior stabilization and interbody reconstruction. The postoperative course was uneventful, and, at the 52-month follow-up, the patient remained neurologically intact with no local or systemic recurrence. This case underscores the significance of precise preoperative assessment and planning for achieving successful oncological and functional outcomes. Although technically demanding, EBR provides excellent local tumor control and durable results in carefully selected patients with solitary spinal metastases. The long-term disease-free survival in our patient highlights the potential value of this treatment for comprehensive metastatic spine management. Continued research and multicenter collaboration are necessary to standardize the criteria for patient selection, refine the surgical techniques, and establish evidence-based guidelines for the use of EBR to treat metastatic spinal tumors.

## Introduction

Solitary spinal metastases are increasingly identified thanks to advances in imaging, and this identification has prompted evolution in the strategies for managing this form of malignancy. The treatment options for solitary spinal metastasis include stereotactic radiotherapy for the precise control of local tumors, surgical decompression and stabilization for neurological compromise or instability, systemic therapy (targeted or hormonal chemotherapy) for disease control, minimally invasive procedures such as vertebroplasty or ablation for pain and support, and supportive care with steroids, analgesia, and bone-strengthening agents [1]. En bloc spondylectomy resection (EBR) may be considered in select cases to achieve tumor-free margins and improve outcomes. However, its role in metastatic spine disease remains controversial and is not considered standard of care. Existing evidence supporting EBR in metastatic settings is limited and largely derived from small case series involving highly selected patients [2]. Here, we present a case of a solitary metastasis to a thoracic vertebra that was successfully managed with EBR. 

## Case presentation

A 49-year-old female with a history of adenoid cystic carcinoma of the nose that had been uneventful for two years presented with a one-week history of back pain. Physical examination revealed no significant findings except for nominal left-side paraspinal muscle spasm in the lower thoracic region. Conventional X-rays and magnetic resonance imaging (MRI) confirmed an eccentric lesion involving the left part of the vertebral body and the left pedicle of the T11 vertebra. A positron emission tomography-computed tomography (PET-CT) scan identified a solitary lesion, and a biopsy confirmed metastatic disease. The patient gave her informed consent to participate in the study.

**Figure 1:**
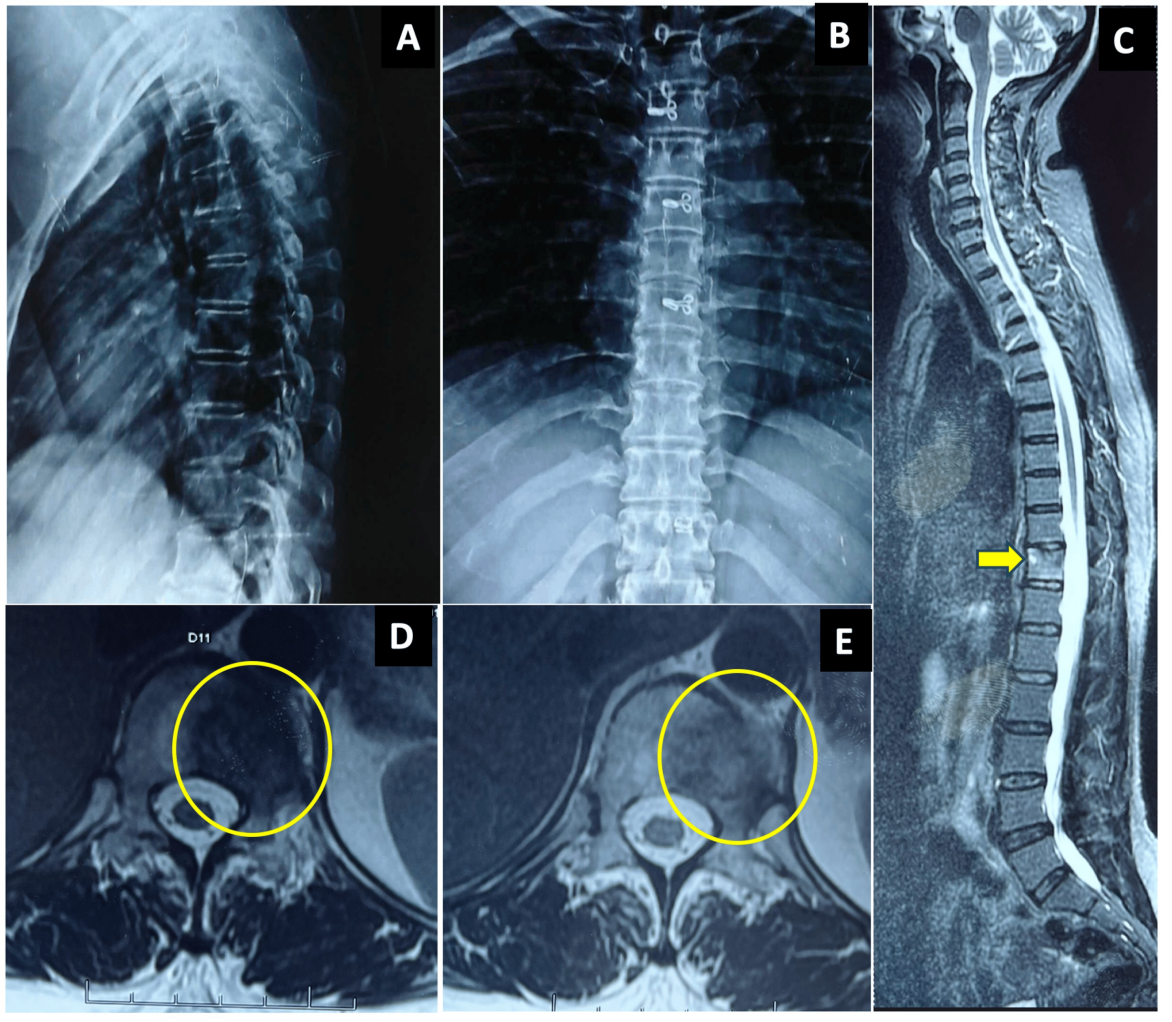
Preoperative radiographs of the patient. The patient’s antero-posterior radiograph (A) and lateral radiograph (B) show no significant findings. The MRI images show sagittal cuts (C) with the T11 tumor (yellow arrow) and MRI axial cuts (D, E) with the tumor inset (yellow circle). MRI: magnetic resonance imaging

**Figure 2:**
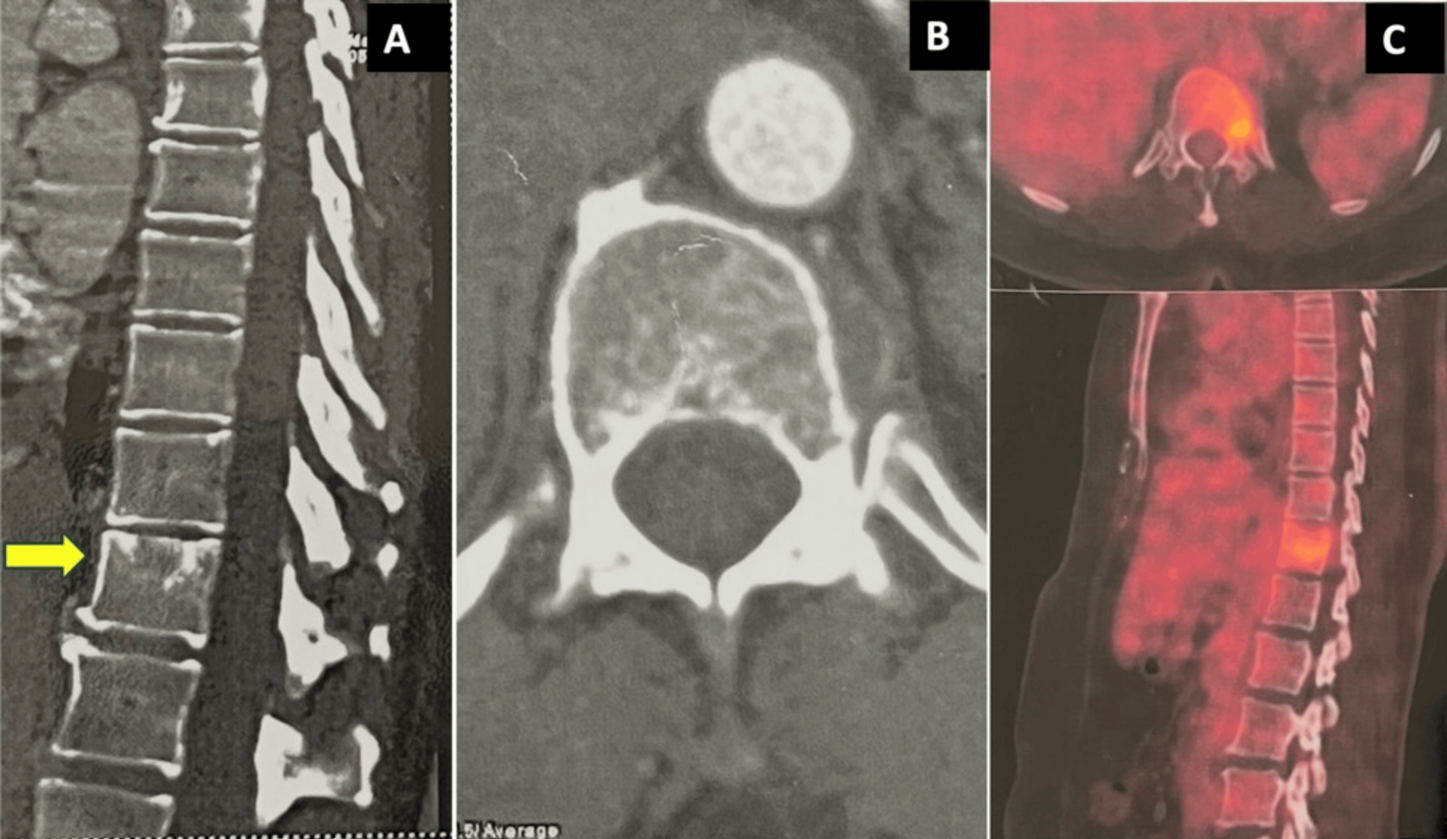
Preoperative CT and PET scans of the patient. The CT scan shows the mid-sagittal cut (A) with the T11-involved vertebra (yellow arrow) and the axial cut (B) of the involved vertebra without any significant findings. The PET-CT scan (C) shows increased uptake in the index vertebra. CT: computed tomography; PET: positron emission tomography

The preoperative X-rays and MRI results are shown in Figure 1, and the preoperative CT and PET scan results are shown in Figure 2.

**Figure 3:**
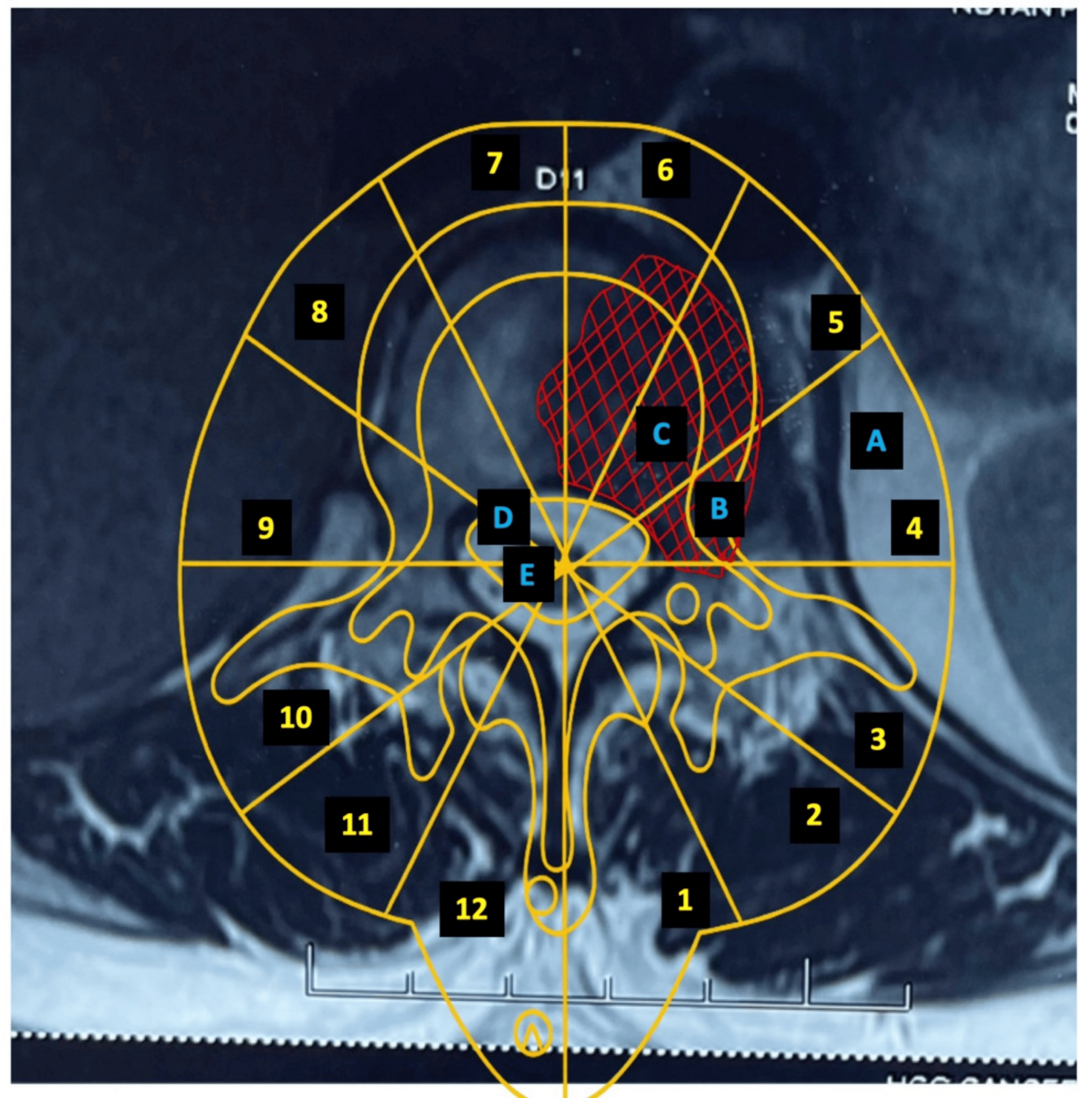
MRI axial cut of the involved vertebra. The tumor-affected region is marked with red hatching. The WBB classification outline (thoracic and lumbar) overlapped the MRI to identify the various radiating sectorial zones (indicated by the counterclockwise yellow numbers) and the concentric layers of involvement (indicated by the blue letters). The diagram makes clear that the involved regions were sectors 4 to 7 (including the pedicle) and layers B and C. MRI: magnetic resonance imaging; WBB: Weinstein-Boriani-Biagini

Because further systemic evaluation showed no evidence of widespread disease, the patient was an ideal candidate for EBR. Considering the solitary nature of the metastasis, EBR was planned to achieve tumor-free margins and prevent recurrence. The Weinstein-Boriani-Biagini (WBB) classification was used to determine the best approach [3]. This case involved sectors 7 to 4 and layers B and C. Figure 3 shows the preoperative axial cuts depicting the involved regions according to the WBB classification.

While the patient was under general anesthesia and intraoperative neuromonitoring, the standard posterior midline approach was taken. The surgical intervention began with posterior stabilization using a pedicle screw-rod construct spanning two adjacent levels (eight screws). Pedicle screws were inserted with the assistance of three-dimensional CT scans. The left and right sides were approached sequentially, with temporary stabilization using a contralateral rod. The posterior elements of the T11 vertebra, that is, the lamina along with the spinous process, were excised with an ultrasonic bone scalpel (BoneScalpel™, Misonix, Inc., NY, USA), and then piecemeal flavum resection was completed. The bilateral roots of T11 were sacrificed, and the left-sided pedicle was spared. A 5-cm segment of the T11 and the rib head was excised bilaterally. Blunt digital bilateral retro-pleural dissection was performed anteriorly. A Gigli’s saw wire was used to complete the discal controlled cuts up to the posterior longitudinal ligament (PLL) while preserving the neural structures. The PLL was cut with a sharp knife. The T11 vertebra was removed as a specimen by rotating it around the dural sac and delivering it out from the left side.

**Figure 4:**
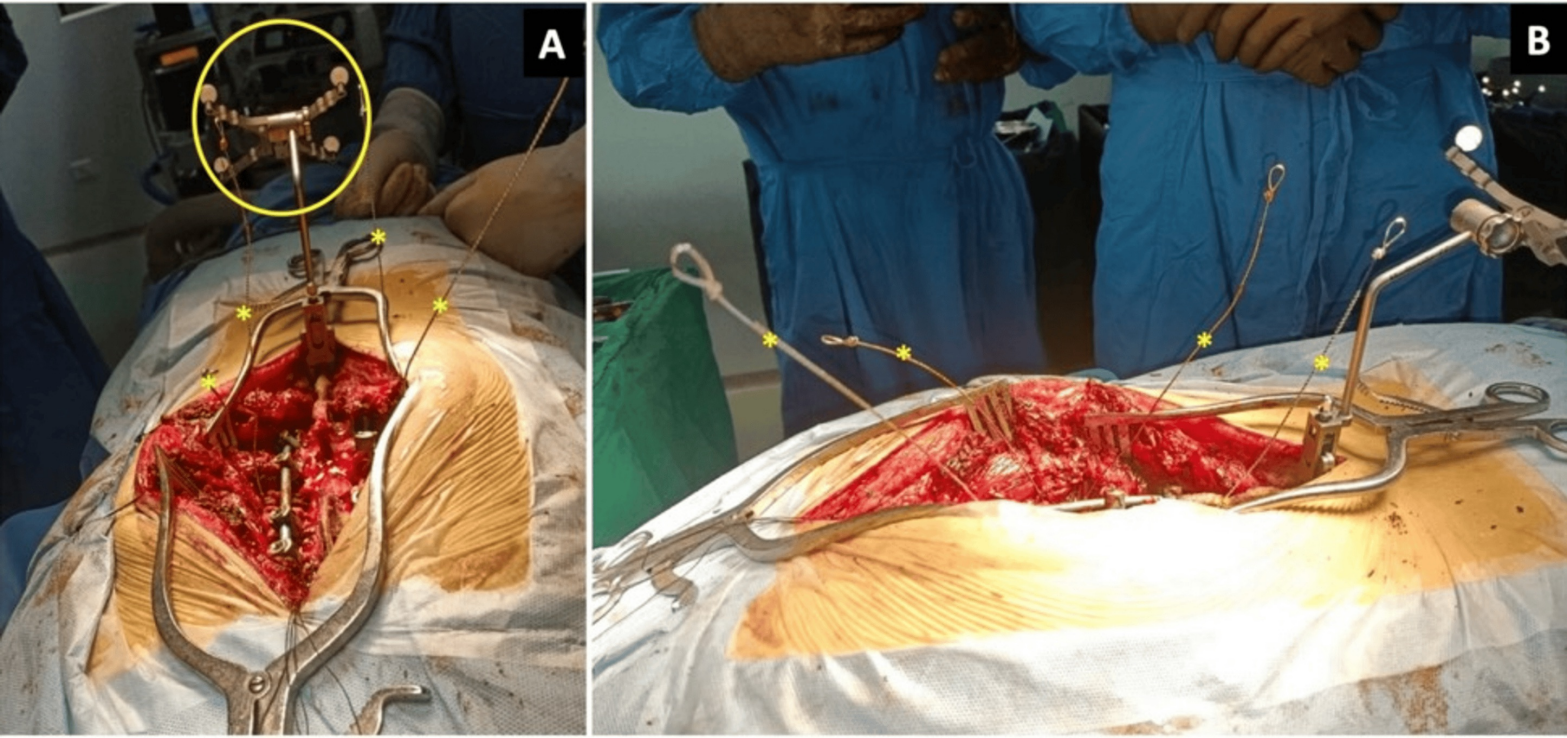
Intraoperative image taken just before the cranial and caudal cuts were made to free the T11 vertebra from the adjoining vertebrae. (A) The procedure guided by three-dimensional computed tomography navigation with the reference frame at the caudal end of the spinous process (yellow circle). A temporary stabilizing rod was inserted, and the Gigli’s saws were arranged to encircle the cranial and caudal disc of the involved T11 vertebra (the four ends of the two saw wires are marked with asterisks). (B) Lateral image showing the cranial and caudal arrangement of the two Gigli saw wires.

**Figure 5:**
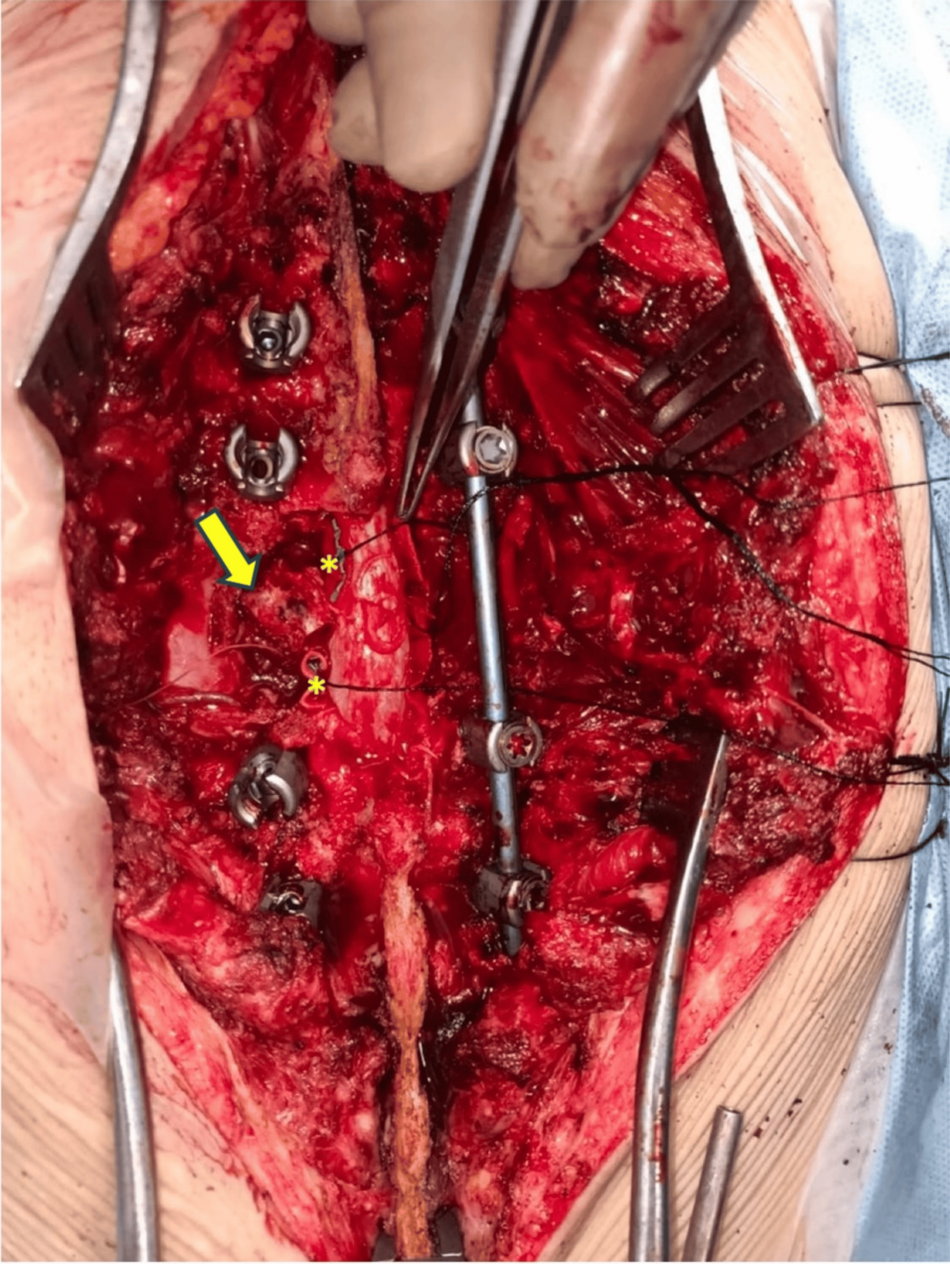
Image taken after the en bloc removal of the lamina showing the adjoining ligamentum flavum. The en bloc resection was done with an ultrasonic bone scalpel. The right pedicle was also removed. The disc cuts were completed with Gigli saw wires. The posterior longitudinal ligament was cut at both discal levels. The involved pedicle (marked with a yellow arrow) was left unintervened. Neurosurgical patties (the yellow asterisks) served to protect the cord. The vertebra was rotated and delivered out carefully through the left side.

Figure 4 shows intraoperative images during the use of Gigli’s saw. Figure 5 is a post-laminectomy image showing the exposed pedicle at the affected level.

Anterior column reconstruction was carried out using an expandable cage, with posterior bilateral fixation of the final rods. Interbody bone grafting was performed using local cancellous bone. Additionally, an inter-transverse rib bone graft was used as an accessory strut on the left side between the T10 and T12 transverse processes.

**Figure 6:**
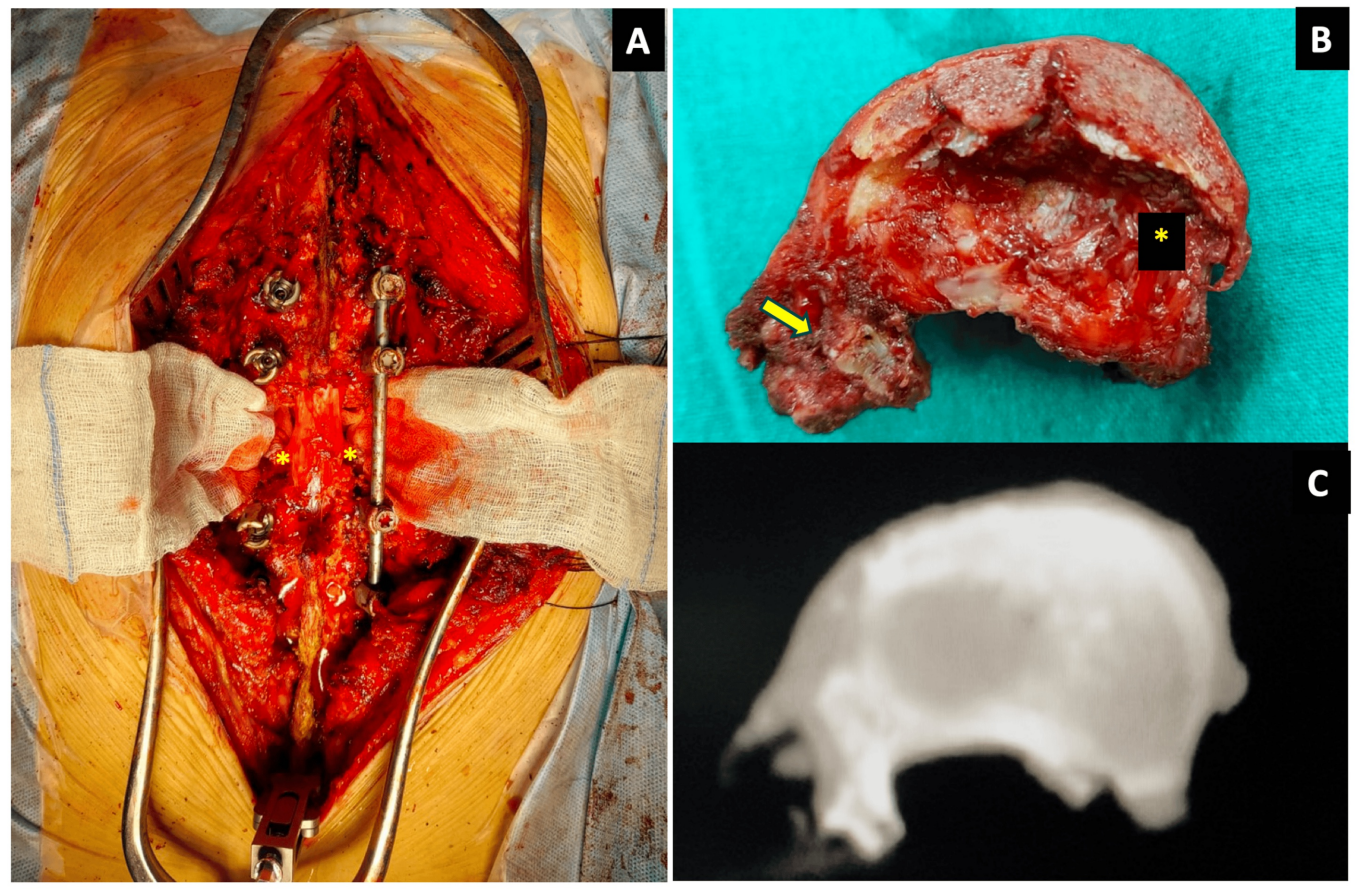
Images after the delivery of the vertebra. (A) A gauze piece was inserted under the cord to increase the visibility of the bilateral sacrificed roots (marked by two yellow asterisks). The through-and-through gap created by the en bloc resection of the T11 vertebra is apparent. The T11 roots had to be sacrificed (the two yellow asterisks on either side mark the stumps of the ligated roots). (B) Specimen picture of the en bloc excised T11 vertebral body from above showing the cranial disc side (marked by a yellow asterisk) and the involved pedicle (marked by a yellow arrow) removed en bloc. (C) The correlating CT scan of the en bloc resection specimen confirmed the margin of excision radiologically.

Following en bloc vertebral excision, postoperative CT imaging confirmed both complete excision of the lesion and the accuracy of screw placement. Histopathological analysis confirmed negative margins and metastatic adenoid cystic carcinoma. An intraoperative image following delivery of the vertebra is shown in Figure 6.

**Figure 7:**
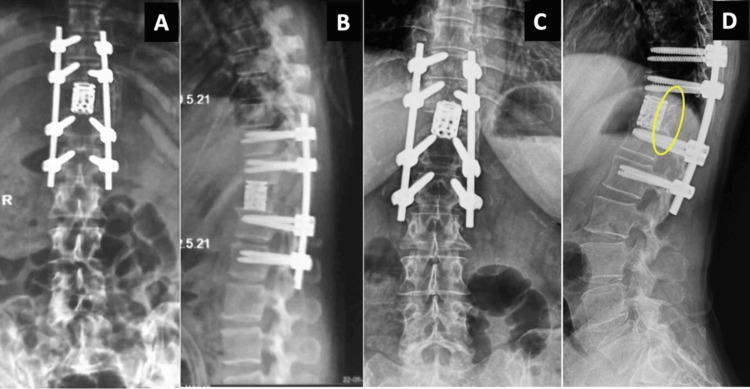
Postoperative radiographs. Immediate postoperative views (A, B) show a pedicle-screw construct spanning two levels above and below, with anterior column reconstruction using an expandable cage. Radiographs at the four-year follow-up (C, D) show that the implant position was well-maintained, as well as nominal cage subsidence and the bridging callus (yellow circle) on the posterior-lateral left side of the cage.

Closure under the drain was performed, and the patient recovered well, with no complications. Mobilization was immediately achieved, and rehabilitation was started at three weeks. In view of the solitary nature of the metastasis, complete en bloc excision with negative margins, absence of residual local disease, and lack of evidence of systemic spread on PET-CT, adjuvant radiotherapy or systemic therapy was not administered after multidisciplinary discussion. Follow-up imaging at four months showed that the reconstruction and spinal alignment remained stable. At 52 months, there was no evidence of recurrence, and interbody union was achieved. Figure 7 shows the postoperative radiographs.

## Discussion

Cancers of the nose and paranasal sinuses are rare, accounting for less than 1% of all malignancies [4]. Among these cancers, adenocarcinomas, including adenoid cystic carcinoma, are the third most common mucosal malignancy after squamous cell carcinoma and adenoid cystic carcinoma, representing approximately 15% of all sinonasal cancers [4]. Surgery remains the mainstay of treatment for sinonasal adenocarcinoma, providing the best chance of local tumor control, symptom relief, and improved long-term outcomes [5]. This cancer carries substantial metastatic potential, with studies showing that 15.4% of cases developed metastatic disease, and distant metastases occurred in 12.8% of patients, predominantly spread hematogenously, with the skeletal system, especially the spine, being the most common site, followed by the lungs and the liver [6]. As the researchers observed, the high prevalence of spinal involvement underscores the need for a proactive treatment approach.

Given the risk of progression and potential for neurological compromise, surgical interventions such as EBR play a critical role in managing spinal metastases, ensuring local tumor control, and improving patient outcomes [6]. Building on their previous studies, in which they identified a few indications for EBR, namely, the absence of widespread metastasis, solitary metastasis confined to the vertebra (i.e., no paraspinal spread), and biologically favorable tumors, Tomita et al. described a surgical strategy for spinal metastasis using a score to decide between excisional and palliative procedures [7]. This score takes into account prognostic factors such as primary tumor treatability, visceral metastases, and the number of bone metastases. They recommended wide or marginal excision for cases with EBR in cases with a prognostic score of 2 or 3, while marginal or intralesional excision is preferred as the score reaches 4 or 5; palliative surgery is advised for scores of 6 or 7, and only supportive care is required for scores of 8, 9, or 10 [8].

Total EBR for solitary spinal metastasis has been studied by multiple authors. In their mean follow-up of 15 patients at 33 months, Melcher et al. found no recurrence in 11 patients, while four had a recurrence of the disease [9]. In a study by Zhou et al. comparing total en bloc with piecemeal excision, 60 patients with Enneking stage III giant cell tumors had better outcomes with EBR [10]. En bloc surgeries in cases with well-confined tumors, such as intra-compartmental cases, have a better prognosis with EBR than cases with paraspinal spread (extra-compartmental). In a case series of 12 patients, Sakaura et al. found recurrences in two of the four cases with paraspinal spread, whereas the cases without spread had no recurrence [2].

The evolution of spondylectomy techniques ultimately led to the need for a reproducible system that could rationalize surgical planning based on tumor anatomy rather than operative preference alone [11]. The rationalization of the surgical approach was first attempted by Weinstein, Boriani, and Biagini in a study published in 1997 [3]. These researchers divided the vertebral body into 12 equal sectors in an anticlockwise manner and four layers from superficial to deep. They proposed that EBR using posterior and anterior approaches would be appropriate for cases in which sectors 4-9 are involved, at least one pedicle is tumor-free, and there is no invasion of the epidural space. They further proposed that sagittal resection is indicated for cases involving sectors 2-5 or 8-11, while posterior resection is appropriate for cases in which sectors 10-3 are involved [3].

In a 2014 paper on the indications, planning, and morbidity of EBR, Boriani et al. detailed the indications and planning for EBR in the spine. They classified their approaches into types, each with various subtypes. Type 1 includes only anterior approaches and is indicated for tumors involving only the anterior aspect of the body. Type 2 involves various posterior approaches, while Types 3 and 4 require a dual approach (anterior and posterior). More specifically, Type 3 includes anterior release and posterior delivery, while Type 4 shows posterior piecemeal excision followed by anterior excision [12].

In our case, sectors 7-4 were involved, with an eccentric lesion and one pedicle being tumor-free, so the case was appropriate for a Type 2B surgical approach according to the WBB planning. This approach requires the piecemeal removal of the posterior arch that is not affected by the tumor, followed by blunt dissection of the anterior part of the body from the anterior structures and transdiscal detachment above and below, thus allowing the rotation of the excised vertebral body around the cord to deliver it posteriorly.

Though EBR preserves neurological function and structural stability, it is a major procedure associated with a high risk of morbidity. In a 2010 study of the morbidities, Boriani et al. studied 1,135 patients and found infections, hardware failure, wound dehiscence, hematomas, and vascular injuries to be major complications in these procedures [13]. The rate of complications was 34.3%, and these researchers also identified the major risk factors for complications as multi-segmental resections, combined anterior and posterior approaches, and prior radiation therapy or re-operations [13]. However, the long-term benefits of EBR in preventing recurrence outweigh these challenges [9]. In a case series by Boriani et al. involving 220 cases followed for 25 years, published in 2017, the local recurrence rate was 15%, and clear margins were associated with a better prognosis, while the involvement of anterior structures led to poor outcomes [14].

Advances in stereotactic body radiotherapy (SBRT) have significantly expanded the non-surgical treatment options for spinal metastases, offering high rates of local tumor control with reduced morbidity, particularly in patients with stable spines and no significant neurological compromise. In many contemporary oncologic settings, SBRT may be preferable to surgery in patients with limited physiological reserve or those with multifocal disease [15]. However, direct comparative outcome data between SBRT and EBR remain limited, particularly for solitary spinal metastases. Additionally, much of the surgical literature supporting EBR is derived from giant cell tumors or primary spinal malignancies, and extrapolation of these results to metastatic disease must be undertaken with caution [1]. Consequently, EBR cannot be considered a standard of care for spinal metastases; rather, it represents a potential option in exceptional, highly selected cases where complete resection with negative margins is feasible, systemic disease is absent, and long-term survival is anticipated.

Our patient’s 52 months of recurrence-free survival further demonstrate the relevance of EBR in cases of solitary metastatic spine tumors without paraspinal involvement.

## Conclusions

EBR was a feasible and efficacious surgery in this case of a solitary metastasis. Larger case series and multicentric reports could validate the general application of this approach. Technological add-ons such as intraoperative CT navigation, ultrasonic bone scalpel, and intraoperative neuromonitoring can make the approach safer.
